# The Effects of Preparation Methods and Internal Electron Donors on Ziegler-Natta Catalyst Performance and Polypropylene Properties

**DOI:** 10.3390/polym18101214

**Published:** 2026-05-16

**Authors:** Bin Li, Huashu Li, Zhuo Chen, Hongfan Hu, Yi Zhou, Guoliang Mao, Shixuan Xin

**Affiliations:** 1 Provincial Key Laboratory of Polyolefin New Materials, College of Chemistry & Chemical Engineering, The Northeast Petroleum University, Daqing 163000, China; libin14152@163.com (B.L.); maoguoliang@nepu.edu.cn (G.M.); 2 PetroChina Petrochemical Research Institute, PetroChina Company Limited, Beijing 102206, China; lihuashu@petrochina.com.cn (H.L.); huhongfan@petrochina.com.cn (H.H.); zhouyi9@petrochina.com.cn (Y.Z.); 3 Sinopec Maoming Petrochemical Company, Maoming 525000, China; chenzhuo.mmsh@sinopec.com

**Keywords:** Ziegler-Natta catalyst, internal electron donor, isotactic polypropylene, MgCl_2_ support, stereoselectivity

## Abstract

Ziegler-Natta (Z-N) catalysts for propylene polymerization were prepared in situ using dibutyl phthalate (DNBP) or 9,9-bis(methoxymethyl)fluorene (BMMF) as internal electron donors (IDs) by treating the support precursors (Mg(OEt)_2_ or MgCl_2_·2.5EtOH) or MgCl_2_ complex solutions with TiCl_4_ respectively. In this study, eight Z-N catalysts containing two types of IDs were prepared via different preparation routes and systematically characterized with modern analytical techniques. The results indicated that, even with the same IDs, the catalysts prepared by different methods exhibited significant differences in chemical composition, particle size distribution, catalytic activity and stereoselectivity. The properties of polypropylene (PP) were largely influenced by the preparation route of the catalysts. Particularly, the catalysts obtained by the reprecipitation method showed the highest catalytic activity and the smallest MgCl_2_ particle size. The distribution of stereoselective active centers in the catalysts was simultaneously affected by the preparation method and the type of IDs. In addition, the melting point (T_m_) of PP could be used as an effective indicator to evaluate the relative content of the highly isotactic active centers in the catalysts. This study provides valuable insights into the rational design of Z-N catalysts for propylene polymerization, highlighting the critical role of the preparation methodology in tailoring the catalyst properties and active center distribution.

## 1. Introduction

Isotactic polypropylene (iPP) is deeply rooted in the daily life of modern society. iPP is produced in hundreds of millions of tons per annum, and it is applied in various fields such as home appliances, automotive, construction, hygienic goods, food/medical packaging. It appears in the form of films, sheets, tubes, fibers, nonwovens, and other sophisticated objects. The majority of iPP is manufactured using high-performance Ziegler-Natta (Z-N) catalyst systems.

Z-N catalyst consists of three major components: the support (MgCl_2_), the internal electron donor(s), and the core of the active center metal (TiCl_4_). The manufacture of high-performance Z-N catalysts requires support to stabilize the catalytic active centers. Anhydrous MgCl_2_ is widely regarded as the preferred support for Z-N catalysts in olefin polymerization, due mainly to its crystal structure and lattice parameters that are analogous to α-TiCl_3_ active component and closely match the dimensions of Mg^2+^ (0.65 Å) and Ti^4+^ (0.68 Å), and the layered structure of MgCl_2_ (CCP) and α-TiCl_3_ (HCP). Ever since the discovery of the excellent properties of MgCl_2_-supported Z-N catalysts for stereospecific olefin polymerization, extensive research in improving Z-N catalyst performance has been directed toward the development of the novel internal electron donors (IDs), and the supports which possess high specific surface areas and well-defined morphology [1,2,3]. These strategies are designed to improve catalytic stereoselectivity and productivity through the rational design of advanced IDs and the systematic optimization of the catalyst preparation protocols. The overarching goal is to achieve catalyst systems with superior performance through the rational optimization of catalyst architectures, thereby addressing the ever-escalating performance requirements for iPP homopolymer and copolymer materials.

The catalyst preparation procedure appears to be one of the most important factors in determining catalyst performance, as it endows the final polymer with distinct properties. Since MgCl_2_ was adopted as a support in third-generation Z-N catalysts, the catalytic system has undergone extensive development and iterative refinement.

Z-N catalysts employing MgCl_2_ as support can be prepared via mechanical or chemical methods. The mechanical preparation method involves co-milling all components of the main catalyst, thereby simultaneously loading the active species and IDs onto the support and modifying the crystal structure of MgCl_2_ during milling [4,5,6]. The co-milling method facilitates the reduction in MgCl_2_ crystallite size, thereby increasing the catalyst’s surface area and enhancing its activity. However, a significant limitation remains the inherent challenge in controlling polymer morphology.

The procedure for the first type of chemical reaction method begins with the mixing, heating, and dissolution of anhydrous α-MgCl_2_ together with other reactants, typically alcohol-hydrocarbon mixtures or epichlorohydrin (ECH) combined with tributyl phosphate (TBP), to form a solution containing the complex compound [7,8,9,10]. Subsequently, TiCl_4_ is added to the composite solution to initiate the reaction, during which the crystal structure of MgCl_2_ is transformed, and the MgCl_2_-Ti complex precipitates progressively. Another chemical reaction procedure involves using Mg(OEt)_2_ or MgCl_2_·nROH as support precursors, which are dispersed in an organic solvent and subsequently react with TiCl_4_ and IDs to yield MgCl_2_. During the impregnation phase, the precursor reacts with TiCl_4_ in the presence of hydrocarbon solvents, facilitating the reaction within this phase to form MgCl_2_-supported Z-N catalysts [11,12,13]. This process concurrently generates the byproduct species TiCl_4-n_(OEt)_n_, which negatively affect the polymerization activity [14].

Two catalysts were prepared via dissolution-reprecipitation and chemical conversion. Surface titanium content was analyzed by electron spin resonance (ESR). The catalyst prepared by chemical conversion exhibited lower surface titanium content than the one prepared by solvothermal precipitation. This difference is proposed as a contributing factor to its lower catalytic activity [15]. Taniike et al. analyzed an Mg(OEt)_2_ supported Z-N catalyst and found that microscopic structural parameters (e.g., micropore volume and chemical composition) were largely independent of Mg(OEt)_2_ particle morphology. In contrast, catalyst number-average particle size exhibited a negative correlation with ethylene/1-hexene copolymerization activity, while mesopore and macropore volumes correlated positively with 1-hexene insertion efficiency [16].

Malanasi et al. prepared three catalysts via ball milling, chemical conversion, and dissolution-reprecipitation, each incorporating the same 1,3-diether ID. The synthetic method dictated the morphology and size of the MgCl_2_ support particles, with those produced via dissolution-reprecipitation exhibiting the smallest dimensions. This particle size difference consequently influenced the electronic properties and local coordination structure of the titanium active sites. Specifically, titanium sites in catalysts prepared by chemical conversion possessed a higher effective positive charge than those generated by ball milling. The quantity of accessible Ti^3+^ sites, determined via room-temperature CO adsorption infrared spectroscopy, was highest for the sample. Overall, the three activated catalysts demonstrated significantly divergent kinetic behaviors in propylene polymerization. These kinetic characteristics correlated with both the number of accessible Ti^3+^ sites and the effective positive charge of the titanium sites [17].

Reza et al. treated a MgCl_2_·1.5EtOH support with aluminum compounds, yielding a catalyst with an increased specific surface area and a reduced particle size. Density functional theory (DFT) calculations indicated higher adsorption energies, and elemental analysis confirmed the presence of aluminum, suggesting that the aluminum precursors co-incorporated with TiCl_4_ into the catalyst structure. This treatment significantly altered the olefin polymerization kinetics, affecting both the polymerization rate profile and the final polymer’s flow morphology [18]. Zarupski et al. synthesized highly active supported Z-N precatalysts via the reaction of dehydroxylated silica with an organomagnesium compound and TiCl_4_. Complementary techniques—including Ti K-edge XANES, Ti L_2,3_-edge NEXAFS, DR UV-Vis-NIR spectroscopy, and DFT simulations—revealed that the precatalyst comprises both small TiCl_3_ clusters and mixed Mg–Ti oxychloride structures deposited on the silica surface [19]. Further analysis identified a coexistence of α-/β-TiCl_3_-like clusters, monomeric Ti(IV) and isolated Ti(III) with their relative concentrations depending on the alkylaluminum activator concentration. At the exterior of the catalyst particle, TiCl_3_ clusters are preferentially formed, likely due to Ti(III) mobility in the presence of strong Lewis acids, which often obscures the spectroscopic detection of isolated Ti(III) sites. In contrast, only monomeric Ti(III) sites were formed in the particle interior; these were characterized by a high electron density, indicative of the proximity of the electron donors [20]. Yakimov et al. introduced BCl_3_ during catalyst preparation and quantitatively analyzed the relative proportion of Ti sites coordinated by Cl and O-donor ligands. This was achieved by employing a library of theoretical X-ray absorption spectroscopy (XAS) lines, which were computed from DFT-optimized structural models. This approach provides a method to quantify the formation of specific metal species in complex Z-N catalysts and to correlate these species with catalytic activity [21].

However, the heterogeneity of Ti species in Z-N catalysts, the mutual interactions among different components and the high flexibility of the catalytic system have led to difficulties in understanding the effects of different preparation routes and types of electron donors on catalytic performances [22,23]. These main open questions are still offering challenges for the future development of more prominent catalysts. In present work, we used different preparation methods and supported DNBP or BMMF as IDs, respectively, and prepared eight Z-N type catalysts. These catalysts were characterized using all appropriate analytical means, and the information was correlated with their apparent catalytic performance and the relevant properties of iPPs.

## 2. Materials and Methods

All manipulations (polymerization, pre-catalysts preparation, and treatments) were performed using standard Schlenk techniques under a high-purity nitrogen atmosphere.

### 2.1. Materials

Anhydrous MgCl_2_ (99.99%) was purchased from Macklin (Shanghai, China) and subjected to a 4 h grinding process using a ball mill prior to the experimental procedure. Titanium tetrachloride (99.99%, Metal basis) was purchased from Macklin. Triethylaluminum (TEA, 2.0 mol/L in *n*-heptane) was purchased from Energy Chemical (Shanghai, China), used as received without further treatment. Propylene (99.9%, supplied by YHX Gas Co., Zhuhai, China) was purified by passing through columns packed with molecular sieves and Copper-based deoxygen agent. All the regents including *n*-heptane, *n*-hexane, *n*-decane, toluene, 2-ehtyl-1-hexanol (2-EH), tributylphosphate (TBP), epichlorohydrin (ECH), phthalic anhydride (PA), di-*n*-butyl phthalate (DNBP), 9,9-bis(methoxymethyl)-fluorene (BMMF) and cyclohexyl(dimethoxy)methylsilane (CHMMS, external doner) were purchased from Macklin and dried over 4 Å molecular sieves under dry nitrogen prior to use. Mg(OEt)_2_ and MgCl_2_·2.5EtOH were supplied by PetroChina Petrochemical Research Institute, Beijing, China.

### 2.2. Preparation of Pre-Catalysts

#### 2.2.1. Preparation Procedures for Cat-A-1 and Cat-A-2

Cat-A-1 and Cat-A-2 were prepared through the chemical reaction of Mg(OEt)_2_ precursor with TiCl_4_. In detail, Mg(OEt)_2_ (5 g, 0.044 mol) was suspended in toluene under stirring. Subsequently, TiCl_4_-Toluene solution 40 mL(4: 6, *v*/*v*) was syringe-injected dropwise into the suspension at a temperature below 0 °C and the mixture was heated up to 40 °C, and ID (DNBP or BMMF, 0.05 mol) was added. The temperature was allowed to rise to 110 °C and kept for 2 h. The resulting suspension was settled at ambient temperature, and the solid was washed with TiCl_4_-Toluene solution 50 mL (4: 6, *v*/*v*) for four times to remove the by-product. The resulting solid was washed with *n*-hexane for three times. Finally, the Z-N type catalysts, Cat-A-1, and Cat-A-2, were obtained after drying under high vacuum.

#### 2.2.2. Preparation Procedures for Cat-B-1 and Cat-B-2

Cat-B-1 and Cat-B-2 were prepared through the chemical reaction of MgCl_2_·2.5EtOH precursor with TiCl_4_. In details, MgCl_2_·2.5EtOH (5 g, 0.024 mol) was suspended in toluene under stirring. Subsequently, TiCl_4_ (50 mL, 0.456 mol) was syringe injected dropwise in the suspension at below 0 °C and the mixture was slowly heated up to 80 °C, and ID (DNBP or BMMF, 0.05 mol) was added. The temperature was allowed to rise to 100 °C and kept for 2 h. The resulting suspension was settled at ambient temperature, washed with TiCl_4_ for four times to remove any by-product. The resulting solid was washed with *n*-hexane for three times. After drying under vacuum to constant weight, the Z-N type catalysts Cat-B-1 and Cat-B-2 were obtained.

#### 2.2.3. Preparation Procedures for Cat-C-1 and Cat-C-2

Cat-C-1 and Cat-C-2 were prepared through a dissolution-reprecipitation method. First, anhydrous MgCl_2_ (5.0 g, 0.053 mol), toluene (100 mL), ECH (4.2 mL, 0.052 mol) and TBP (13 mL, 0.048 mol) were charged to a thoroughly demoisturized and deoxygenized flask under high purity nitrogen atmosphere at ambient temperature. The reaction temperature was increased to 55 °C and kept for 2 h to ensure that the MgCl_2_ was completely dissolved. Subsequently, PA (1.5 g, 0.010 mol) was added and kept to stir at 55 °C for 1 h. The reaction mixture was allowed to cool, and TiCl_4_ (58 mL, 0.528 mol) was added dropwise at −25 °C. The reaction was slowly heated to 80 °C, the ID (DNBP or BMMF, 0.05 mol) was added at this point and it was stirred at 80 °C for 1 h. The obtained product was filtered and washed with TiCl_4_ for three times at 110 °C, followed by washing with *n*-hexane three times and drying under dynamic vacuum to constant weight; the Z-N type catalysts Cat-C-1 and Cat-C-2 were obtained.

#### 2.2.4. Preparation Procedures for Cat-D-1 and Cat-D-2

Cat-D-1 and Cat-D-2 were prepared through a dissolution-reprecipitation method. First, anhydrous MgCl_2_ (7.0 g, 0.074 mol), *n*-decane (70 mL) and 2-EH (30 mL) were charged to a thoroughly demoisturized and deoxygenized flask under a high-purity nitrogen atmosphere at ambient temperature. The reaction temperature was increased to 130 °C and kept for 2 h to ensure that the MgCl_2_ was completely dissolved. PA (1.67 g, 0.011 mol) was added, and the reaction was allowed to continue for an additional hour. Subsequently, the reaction mixture was cooled down, and TiCl_4_ (100 mL, 0.912 mol) was added dropwise at −25 °C. After the system was slowly heated up to 110 °C, ID (DNBP or BMMF, 0.05 mol) was charged at 110 °C and kept for 2 h at this point. Then, the obtained product was hot-filtered and washed with TiCl_4_ for three times at 110 °C, followed by washing with *n*-hexane for at least five times, and drying under dynamic vacuum to constant weight, and the Z-N type catalysts Cat-D-1 and Cat-D-2 were obtained.

### 2.3. Procedure for Propylene Polymerization

After thorough demoisturizing and deoxygenizing an oven-dried Schlenk flask, filled with a high-purity N_2_, fully displaced with nitrogen and propylene to keep the pressure of the system slightly higher than 0.1 MPa. Subsequently, the purified n-heptane (100 mL) was introduced, followed by the addition of TEA (*n*-hexane solution, 2 mol/L) at 50 °C. After stirring for 5 min, the catalyst granules and ED were introduced into the reaction vessel. Following an hour of reaction at a constant temperature, the reaction was terminated by the addition of a MeOH/HCl solution (7:3, *v*/*v*). The obtained PP were filtered and washed at least three times with excess ethanol and distilled water, respectively, and dried in a vacuum oven at 50 °C for 8 h.

### 2.4. Polymer Fractionation

Each PP sample is separated in two steps and then into three fractions. (i) About 2 g of PP was completely dissolved in boiling *n*-octane. The solution was then cooled to room temperature, and after the polymer was fully crystallized, the *n*-octane soluble fraction (C8-Sol) and the solid fraction were separated by filtration. The *n*-octane soluble fraction (C8-Sol) is the lower isotactic PP part. (ii) The *n*-octane insoluble fraction (solid fraction) was first dried in a vacuum and then extracted with boiling *n*-heptane in a Soxhlet extractor for 6 h. The solid fraction (C7-Ins) was collected from the sample holder, and the boiling *n*-heptane soluble fraction (C7-Sol) was recovered from the solution, the former being the highest isotactic fraction of the PP sample and the latter being the medium isotactic fraction of the PP sample. All three fractions were dried in a vacuum, weighed, and recorded.

### 2.5. Characterization

#### 2.5.1. Scanning Electron Microscopy

The morphologies of the catalyst particle and PP particles were characterized by scanning electron microscopy (SEM) with a Hitachi Regulus 8100 instrument, using acceleration voltage 15 kV. All particles were subjected to Pt sputtering for 100 s before the measurements.

#### 2.5.2. X-Ray Diffraction Study

The X-ray diffraction study of Z-N catalysts was performed on a Rigaku D/MAX-2500 diffractometer (Tokyo, Japan) with a Cu Kα source (λ = 1.5406 Å) in the 2θ range from 5° to 60° and a scanning speed of 5°/min. Catalyst samples were prepared in a glovebox with high-purity argon atmosphere and protected with PET film (2θ = 25°).

#### 2.5.3. Molecular Weight and Molecular Weight Distribution

Polymer weight average molecular weight (M_w_) and molecular weight distribution (MWD) were measured by gel-permeation chromatography (GPC) using an Agilent 1260 Infinity II chromatograph (Santa Clara, CA, USA), eluting with 1,2,4-trichlorobenzene at 150 °C and flow rate of 1.0 mL/min. Narrow MWD polystyrene standards were used for GPC calibration.

#### 2.5.4. Melting Point and Crystallinity

Differential scanning calorimetry (DSC) measurements were performed on a NETZSCH 204 F1 Phoenix instrument (Selb, Germany) under a nitrogen atmosphere. Around 5–8 mg of polymer was precisely weighed in a 40 µL aluminum crucible. The testing conditions of the PP samples were strictly carried out in accordance as Ref [24] with the provisions of the GB/T 19466.3-2004 standard. Each DSC measurement consists of two consecutive heating and cooling circles in the 30–230 °C range at a rate of 10 °C/min. The DSC data (T_m_ value) are collected from the second heating ramp. The crystallinity was calculated based on the standard enthalpy (ΔH_m_) of PP with complete crystallinity (209.0 J/g).

#### 2.5.5. Chemical Analysis

The chemical composition of the catalyst samples was analyzed as follows: the Ti content (wt%) of pre-catalysts was determined by the colorimetric method; 100 mg of sample was dissolved in an H_2_SO_4_/H_2_O/H_2_O_2_ solution; and the absorbance at 410 nm was measured using UV-Vis spectroscopy (Agilent Technologies Cary 60, Santa Clara, CA, USA). The Mg contents (wt%) of pre-catalysts were determined by titration using an EDTA solution of known concentration. The content of ID (wt%) was investigated by GC-MS with a Perkin Elmer Clarus 680 instrument (Waltham, MA, USA). The samples were pretreated with ethyl acetate extraction on a shaking bed oscillator. The analytical conditions for the GC-MS are as follows: the initial extraction temperature at 50 °C for 1 min, followed by programmed 10 °C/min temperature control to 280 °C and kept at a 280 °C constant for 5 min.

#### 2.5.6. Particle Size and Particle Size Distribution

The Z-N type catalysts’ particle size and particle size distribution were characterized with a Malvern Analytical Mastersizer 2000 instrument (Malvern, UK). D_10_, D_50_, D_90_ were defined as the particle diameters at 10%, 50%, and 90% in the cumulative number-based particle size distribution. The relative span factor (RSF) calculated based on Equation (1).

(1) $$relative\;span\;factor=\frac{D_{90}{-D}_{10}}{D_{50}}$$

#### 2.5.7. Nuclear Magnetic Resonance Spectroscopy

A Bruker DMX 600M Ultra-Shield spectrometer (Billerica, MA, USA) was used for ^1^H (600.13 MHz) and ^13^C-NMR (150.90 MHz) spectra measurement, and a standard inverse gate decoupling (IGD) Waltz16 program was used for PP sample ^13^C resonance signal quantification. In a typical ^13^C spectrum measurement, 100–120 mg of PP sample were dissolved in 0.50 mL of deuterated *o*-dichlorobenzene-d4 at 393 °K; ordinary *o*-dichlorobenzene was added to provide the internal lock signal at 132.70 ppm, spectral width 200–250 ppm, acquisition time (AQ) 1.5 s, relaxation delay (D1) 10 s, and a minimal of 5000 pulse scans were collected to ensure optimal S/N ratio.

## 3. Results and Discussion

### 3.1. Morphology and Components of Z-N Catalysts

**Figure 1:**
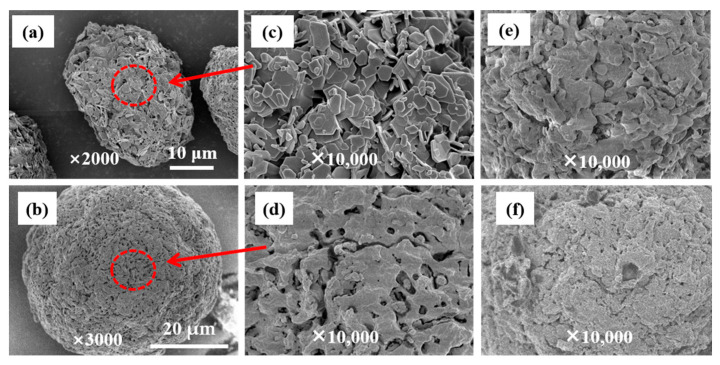
SEM images of the support precursors. (**a**) Mg(OEt)_2_; (**b**) MgCl_2_·2.5EtOH; (**c**) magnified views of Mg(OEt)_2_; (**d**) magnified views of MgCl_2_·2.5EtOH; (**e**) magnified views of Cat-A-1; (**f**) magnified views of Cat-B-1.

Figure 1 displays scanning electron microscopy (SEM) images of two distinct support precursors and catalysts prepared with the two supports in this study. For the chemical conversion preparation route, catalysts fabricated via the in situ reaction between TiCl_4_ and support precursors exhibit a pronounced morphological replication effect, i.e., the catalyst morphology highly preserves the original shape and size characteristics of the precursor particles. Taking the Mg(OEt)_2_ and MgCl_2_·2.5EtOH precursors as examples, SEM analysis reveals that while the macroscopic morphology of the catalysts remains well consistent with their respective precursors, their roughness and pore distribution and the sharpness of the particle edges underwent significant blurring after chemical conversion (Figure 1e,f) [16]. This phenomenon is pervasive across both Cat-A-1/2 and Cat-B-1/2 catalyst samples. Further quantitative analysis demonstrates that the average particle size of the catalysts is slightly larger than that of the support precursors. Combined with local changes in the meso-structure, it can be concluded that although some meso-structural features undergo substantial modifications during the conversion process, the core structural information of the support precursors is still inherited to a measurable extent. This finding provides direct evidence for the conclusion that the chemical conversion method can retain the key structural attributes of the precursors through morphological replication.

**Figure 2:**
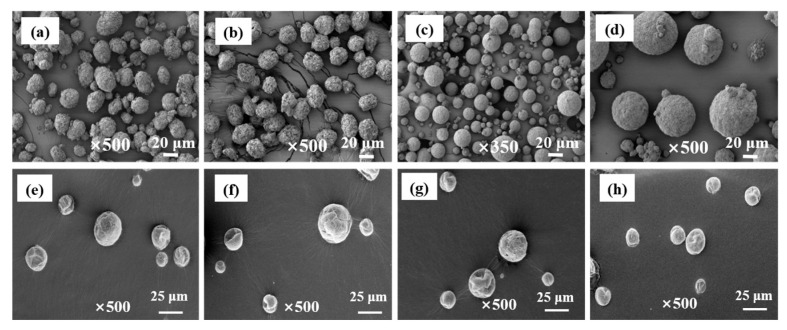
SEM images of the catalysts. (**a**) Cat-A-1; (**b**) Cat-A-2; (**c**) Cat-B-1; (**d**) Cat-B-2; (**e**) Cat-C-1; (**f**) Cat-C-2; (**g**) Cat-D-1; (**h**) Cat-D-2.

Figure 2 displays SEM images of eight distinct catalysts prepared with the different methods in this study. In the dissolution-reprecipitation method, when MgCl_2_ microcrystals precipitate from the mother liquor, their growth process begins at the nucleation center and gradually proceeds through an epitaxial orientation mechanism. Anhydrous α-MgCl_2_, typically prepared by simple ball milling, serves directly as the support precursor and it is subsequently converted into a more disordered, activated MgCl_2_ support via a series of controlled chemical reactions. Titanium and an ID are then loaded in situ onto this support to yield the final catalyst particles, whose morphology is primarily governed by the stirring speed and the molar ratio of the reactants [25]. The primary growth direction of MgCl_2_ microcrystals is longitudinal stacking. Through the stacking of layered MgCl_2_ structures, long rod-shaped crystals are formed, with lengths potentially reaching the radius of primary particles. The Cl–Mg–Cl layers are oriented perpendicular to the rod length, giving rise to a sea urchin-like morphology [26]. In contrast, transverse expansion is constrained by the adsorption of TiCl_4_ onto Mg layers on the lateral surfaces of the layered structure, leading to a smaller transverse dimension (approximately 30–40 Å), which is only 0.1–0.05 times the diameter of the MgCl_2_ rods [27]. Each primary catalyst particle is a spherical particle with a diameter of 5–8 μm and is tightly filled with long crystal rods of MgCl_2_. Growing primary MgCl_2_ particles fuse at their surface, resulting in the formation of tight agglomerates, the final catalyst particles. Furthermore, since ID preferentially adsorbs onto the lateral surfaces of MgCl_2_ crystal rods, it plays a critical role in inhibiting the fusion of rod-like structures via lateral co-crystallization [26].

**Figure 3:**
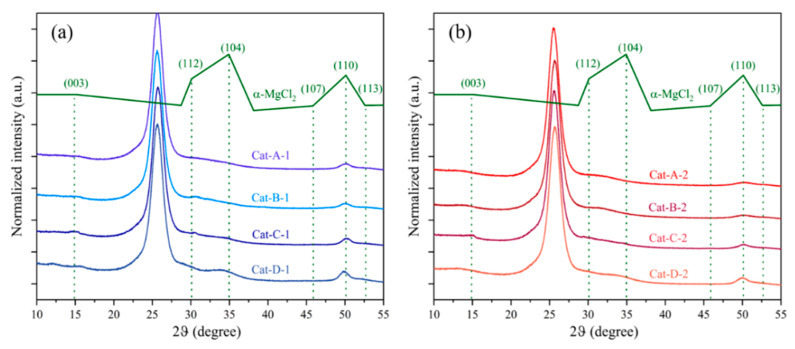
XRD patterns of Z-N catalysts with DNBP ID (**a**) and BMMF ID (**b**). The diffraction pattern of α-MgCl_2_ and the assignment of diffraction planes are also provided as reference (green).

The XRD patterns of the Z-N type catalyst samples are shown in Figure 3. For reference, the pattern of α-MgCl_2_ is shown together with the assignment of corresponding Bragg reflection planes [28]. α-MgCl_2_ has a cubic close packing (CCP) structure, which gives strong diffraction peaks at 2θ = 15° ((003) plane), 30° ((112) plane), 35° (104) plane) and 50° ((110) plane), and the PET film, which gives diffraction peaks at 2θ = 25°, was used to protect all samples from air and moisture contamination [29,30]. Comparative analysis with the standard α-MgCl_2_ PDF database indicates that the crystal structure of the MgCl_2_ support in the catalysts experienced significant modifications. The preparation method exerted a more pronounced influence on alteration of the crystalline form compared to the type of ID. Specifically, the diffraction peaks corresponding to the (003), (112), (104), and (110) planes of MgCl_2_ in the catalyst samples showed noticeable broadening—a trend consistently observed in both the chemical conversion method and the dissolution–reprecipitation method. That is, the representative diffraction peaks became even broader ((104) and (110) planes), while the lower-intensity peaks ((107) and (113)), which were visible in the pattern of all samples, disappeared completely. For Cat-A-1 and Cat-A-2 based Mg(OEt)_2_ support prepared by the chemical conversion method, it is already known that Mg(OEt)_2_ is converted to MgCl_2_ by reaction with TiCl_4_. For Cat-D-1 and Cat-D-2 prepared via the dissolution–reprecipitation method, the diffraction peak at 2θ = 55° exhibited significantly higher intensity, suggesting an increased relative abundance of the (110) plane within the MgCl_2_ support. The extent of structural modification in the MgCl_2_ crystalline support follows this order: (1) By preparation method: Chemical conversion method > Dissolution-reprecipitation method; (2) By ID: BMMF > DNBP. This result indicated that these distinct methods could result in the same catalyst crystal structure.

Eight kinds of catalysts were prepared while changing preparation methods and amounts of two IDs, DNBP and BMMF. Table 1 shows the results of compositional analysis of the prepared catalysts.

Firstly, it demonstrated that catalysts with DNBP ID exhibit lower Ti content than those with BMMF ID [31]. Furthermore, a comparison of the ID content in the catalysts demonstrated that DNBP is higher than that of BMMF when both are prepared using identical procedures. Furthermore, a systematic analysis of the relationship between Ti content and the concentration of the ID across various preparation methods demonstrated that, regardless of whether BMMF or DNBP was employed, the Ti content in the catalyst consistently exhibited an inverse correlation with the donor concentration. This trend was reproducibly observed under all preparation conditions. However, distinct underlying mechanisms are responsible for these observed differences. In the case of the BMMF, it selectively coordinates with Mg atoms on the MgCl_2_ support and does not engage in coordination with Ti species. Consequently, the relative contents of Ti and BMMF in the catalyst are primarily determined by the molar ratio of BMMF to MgCl_2_ introduced during the preparation process [32]. In contrast, for the DNBP, competitive adsorption occurs between DNBP and TiCl_4_ on the MgCl_2_ support surface, where DNBP exhibits stronger coordination affinity toward Mg atoms located on the (110) plane [33]. As a result, DNBP preferentially occupies active sites, limiting TiCl_4_ adsorption to only those regions of the support surface that are sterically inaccessible to DNBP, thereby impeding effective coordination between Ti and the support [34]. Therefore, the trend of relative content of Ti and ID was contrary. In the case of the catalysts (Cat-A-1/2, Cat-B-1/2, Cat-C-1/2 and Cat-D-1/2) which were prepared using different methods respectively, the Mg contents were found to be similar to each other. Furthermore, no significant differences were observed between catalysts containing the same ID (18.67% vs. 17.46%, 17.65% vs. 16.88%, 19.12% vs. 19.79%, and 17.21% vs. 17.78%).

### 3.2. Z-N Type Catalytic Performance in Propylene Polymerization

The results of propylene polymerization for the prepared catalysts are summarized in Table 2. The polymerization data of Ref-1 was obtained from reference [35]. The evaluation of Ref-1 was conducted by bulk polymerization of propylene, and the II% of PP was expressed by the xylene solubles. With a focus on catalytic activity, the catalysts with BMMF ID exhibited higher activity compared to those with DNBP ID. Under the condition of having an identical ID, the impact of the preparation methods on catalytic activity is an obvious distinction. Cat-D-2 prepared with the dissolution-reprecipitation method showed the highest catalytic activity. Catalysts prepared with the dissolution-reprecipitation method exhibit higher activity, which is attributable to two factors: (i) the MgCl_2_ crystallites obtained by this method are noticeably smaller than those from the chemical conversion method; the reduced crystallite size suppresses electron density transfer from the MgCl_2_ surface to TiCl_4_ species, increasing the positive charge at Ti^4+^ sites and thereby promoting olefin monomer insertion to enhance intrinsic catalytic activity [17,36]; and (ii) the dissolution-reprecipitation method exhibited higher surface Ti loading compared with the chemical conversion method, and the lower surface loading of Ti species in the chemical conversion method limited accessibility of embedded Ti species for conversion into catalytic active sites [15].

**Figure 4:**
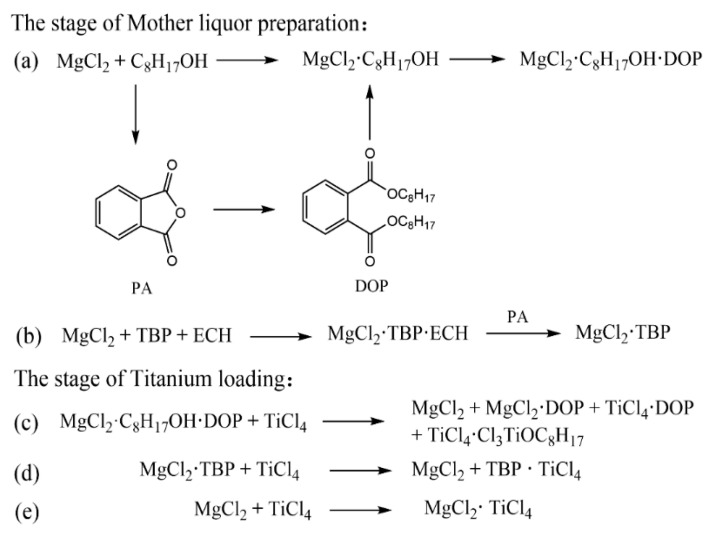
The two different chemical reaction pathways in the dissolution-reprecipitation method. Two Preparation Methods of MgCl_2_ Mother Liquor (**a**,**b**); The precipitation process of activated MgCl_2_ (**c**,**d**); The in-situ loading process of TiCl_4_ (**e**).

In the dissolution-reprecipitation method, both MgCl_2_/ECH/TBP and MgCl_2_/decane/2-EH systems shared a defining feature: MgCl_2_ microcrystal precipitation from the mother liquor is intrinsically coupled with titanium loading. But their reaction mechanisms are fundamentally divergent (Figure 4) [26,37,38]. First, during mother liquor preparation, MgCl_2_/ECH/TBP forms the well-defined ternary complex MgCl_2_·ECH·TBP; adding phthalic anhydride (PA) triggers selective MgCl_2_ precipitation (Figure 4b). In contrast, MgCl_2_/decane/2-EH first forms the binary adduct MgCl_2_·2-EH, with decane acting only as an inert dispersant. PA then reacts in situ with excess 2-EH to generate dioctyl phthalate (DOP), converting the adduct into the thermodynamically favored ternary complex MgCl_2_·2-EH·DOP, and DOP functioning as an effective internal electron donor, comparable to DNBP (Figure 4a). Second, during titanium loading, TiCl_4_ preferentially coordinates with TBP (a stronger σ-donor ligand than MgCl_2_) in the MgCl_2_/ECH/TBP system, displacing MgCl_2_ from MgCl_2_·TBP and forming TiCl_4_·TBP—driving simultaneous MgCl_2_ precipitation and Ti incorporation. In MgCl_2_/decane/2-EH system, TiCl_4_ displaces labile 2-EH ligands from MgCl_2_·2-EH·DOP, triggering ligand dissociation and structural reorganization into a highly porous, defect-rich MgCl_2_ morphology. This yields an activated support with increased surface area and a higher density of coordinative unsaturated Mg^2+^ sites enabling direct TiCl_4_ coordination to unsaturated Mg^2+^ sites on the (100) and (110) planes via chlorine bridges and the formation of stable, surface-bound Ti–Mg–Cl bridged complexes—the catalytic active centers.

The M_w_/M_n_ ratio of PP prepared by single active center catalysts is narrow (2~3) and the wider distribution of molecular weight indicated the catalyst contained multiple active centers. Table 2 listed the molecular weight and molecular weight distribution of PPs obtained from the above-prepared catalysts. The results show that the molecular weight distribution range of PPs prepared with DNBP ID is 6.83 to 7.68, which is wider than that of PPs prepared with BMMF ID. The weight-average molecular weight of PPs prepared using the BMMF ID was lower those prepared using the DNBP ID. Moreover, the difference in preparation methods had little effect on the molecular weight of PPs. The main reason for this situation is that the PP chains produced by BMMF ID catalysts are easier to transfer [2,39].

The dissolution-precipitation method employs magnesium salts, typically anhydrous magnesium chloride, as carrier precursors. These salts initially form stable complexes with various compounds. Following in situ chlorination, the complexes precipitate to form a magnesium chloride support, which is subsequently used to prepare the supported catalysts. Catalysts prepared via this method are characterized by small particle size, high activity, and favorable stereoselectivity. In contrast, the chemical conversion method obtains MgCl_2_ supports through the in situ chlorination of magnesium-containing precursors. This approach yields supports with relatively regular morphology and a controllable pore structure, and with particle sizes intermediate to those produced by ball milling and dissolution-precipitation methods.

### 3.3. Active Center with Different Stereoselectivity

**Figure 5:**
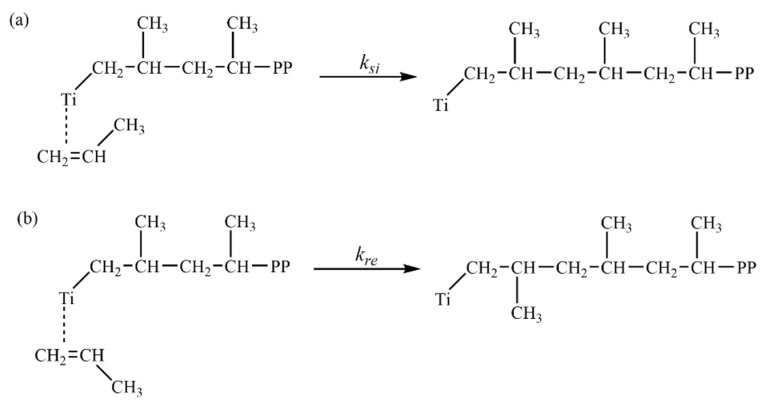
Two potential pathways in the propylene chain growth mechanism: (**a**) *si*-coordinated; (**b**) *re*-coordinated.

A comprehensive model describing the enantioselective active center in Ti-based polymerization catalysts was first proposed by Corradini and became a widely accepted and common model for both heterogeneous and homogeneous olefin polymerization systems [40,41,42,43,44]. In this model, the Ti center contains a coordination vacancy that allows propylene to coordinate in two distinct spatial orientations—referred to as *si* and *re*—based on its alignment relative to the preceding C–C bond in the growing polymer chain, specifically the (Ti)–CH_2_–CH linkage. Upon insertion of the *si*-coordinated propylene into the Ti–CH_2_ bond (Figure 5a), a *meso* linkage is formed with the previously incorporated monomer unit, thereby facilitating continued *isotactic* chain propagation. In contrast, *re*-coordination (Figure 5b), which is sterically disfavored, results in a *racemic* linkage between adjacent propylene units, introducing a stereochemical defect into the polymer microstructure.

If an active center is highly isospecific, *re*-coordination occurs rarely and is corrected in the next monomer insertion step. In such cases, all the stereochemical defects are isolated, and the polymer chains can be described as “mmmmrrmmmm” sequences. Table 3 summarized the stereo sequence distribution data of different types of PP prepared using four types of catalysts. The *meso* sequence length (MSL) is calculated using Equation (2) [2].

(2) $$\;\;MSL=\frac{mmmm+3\times \frac{1}{2}\times mrrr+2rmmr+\frac{1}{2}rmrm+\frac{1}{2}rmrr}{\frac{1}{2}mmmr+rmmr+\frac{1}{2}rmrm+\frac{1}{2}rmrr}$$

Analysis of the data presented in Table 3 reveals that the PP produced with catalysts containing BMMF (Cat-A/B/C/D-2) displays comparatively higher stereoregularity, as determined by both the content of the *isotactic* pentads [mmmm], derived from the ^13^C-NMR data of PP samples, which were obtained by Soxhlet extraction of PPs with boiling n-heptane. The results confirmed that the type of ID and the preparation methods could endow significant impacts on the relative content and distribution of stereo-defects in PP chains. Cat-B-2 has the highest stereoregularity, with [mmmm] content reaching 94.15% and *isotacticity* of 98.85%, the highest among all samples. Further analysis of the ^13^C-NMR spectra of PPs led to two key conclusions: (i) In case of BMMF ID, the pentad sequences [mmmr], [mmrr], and [mrrm] in the resulting PPs exhibited an approximate molar ratio of 2:2:1 [45]. This observation indicates that polymer chain propagation is primarily governed by the stereochemical active center, rather than by chain-end mechanisms. (ii) Across all PP samples, the sequence distributions consistently satisfied the inequalities [mmmm] > [mmmr] and [rrrr] > [mrrr], suggesting a higher propensity for isolated *syndiotactic* placements within predominantly isotactic chains. Taken together, these findings demonstrate that the MgCl_2_/TiCl_4_/ID (DNBP or BMMF)–AlEt_3_/ED catalyst system predominantly forms two distinct types of active centers: *isotactic*-specific and *syndiotactic*-specific.

**Figure 6:**
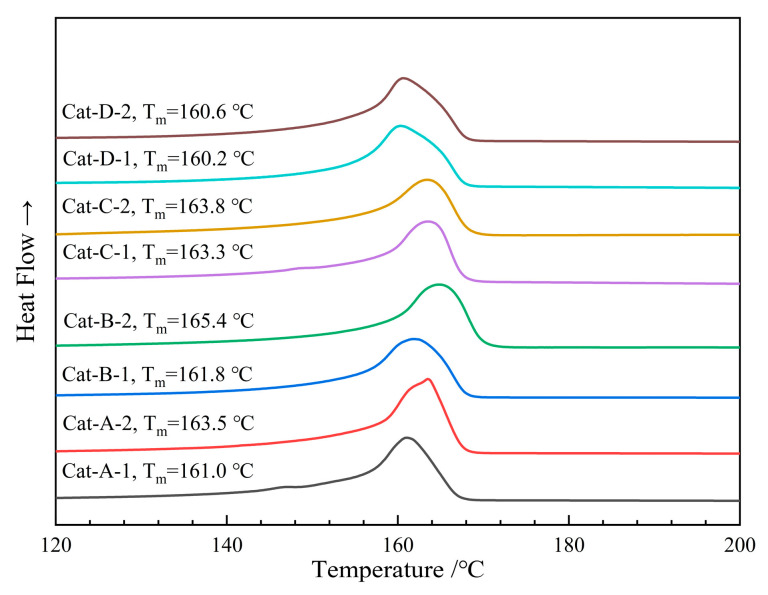
DSC curves of the all-PP samples prepared using Ziegler-Natta catalysts synthesized by different methods.

DSC melting curves of the PP samples are shown in Figure 6. The DSC melting curves reveal that all PP samples exhibit a melting point (T_m_) exceeding 160 °C, with crystallinity values surpassing 46% (determined by normalizing the sample melting enthalpy against a reference value of 209 J/g). A comparative analysis of the T_m_ among different PP samples reveals that both the catalyst preparation method and the nature of the ID significantly influence the melting behavior. The influence of IDs on the melting temperature was further examined: in the DNBP ID cases, the T_m_ difference among PP samples prepared reaches 2.1 °C; in contrast, in BMMF ID cases, this difference increases to 4.8 °C. Moreover, PPs produced with BMMF ID exhibit higher T_m_ than their DNBP ID-based counterpart. Notably, the sample produced using the Cat-B-2 catalyst exhibits both the highest melting point (165.4 °C) and the highest crystallinity (50.4%).

The T_m_ of PP is directly correlated with its stereoregularity, often quantified by the isotactic pentad fraction [mmmm]. Multiple studies confirm that Tm exhibits a positive correlation with the [mmmm] content, reaching maximum values (e.g., T_m_ ≈ 169.9 °C, ΔH_m_ ≈ 104.2 J/g) at high isotacticity (ca. 0.9996) [46,47,48,49]. This relationship stems from the crystallization mechanism: a fully ordered, stable 3_1_ helical conformation—a prerequisite for crystallization—can only form when isotactic sequences within the polymer chain are sufficiently long. Chain propagation errors, such as 2,1-insertions, introduce non-isotactic units. These interruptions shorten isotactic block lengths, reduce the thickness of crystalline lamellae, weaken intra-crystalline van der Waals forces, lower lattice energy, and, consequently, decrease both the melting enthalpy and the observed T_m_. Consequently, the Tm and the width of the melting transition interval in DSC curves reflect the uniformity of the polymer’s stereoregularity. As Kissin et al. demonstrated, multiple melting peaks in PP from multi-site Z-N catalysts originate from components with differing isotacticities [50,51]. A sharp melting curve indicates a uniform population of highly isospecific active sites, whereas broader transitions signify stereoregularity and heterogeneity.

**Figure 7:**
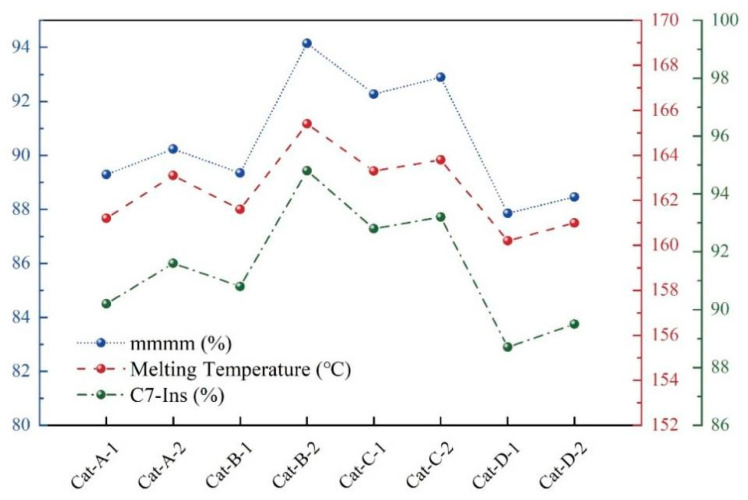
The relationship among [mmmm] (Blue),T_m_ (Red) and C7-Ins components (Green).

Applying this understanding, PP samples synthesized with a catalyst employing BMMF as an ID exhibited a higher T_m_ than those using DNBP. Notably, the sample from catalyst Cat-B-2 possessed the longest isotactic sequence length (MSL) and the highest T_m_. Fractionation studies further link thermal properties to active center distributions. Components such as C8-Sol, C7-Sol, and C7-Ins correspond to *atactic* (aPP), medium-*isotactic* (miPP), and *isotactic* (iPP) polymers, respectively, reflecting the distribution of *atactic*, low-/medium-*isotactic*, and high-*isotactic* active centers in the catalyst [52,53]. In this work, combining fractionation with ^13^C-NMR and DSC data revealed a positive correlation among the content of the high-*isotactic* fraction (C7-Ins), the [mmmm] pentad content, and the sample T_m_ (Figure 7). In summary, the depression in T_m_ is directly attributable to the shortening of *isotactic* sequences caused by stereochemical errors during polymerization. Therefore, a comprehensive analysis integrating DSC melting behavior with the content and distribution of *isotactic* sequences provides a robust framework for evaluating PP stereoregularity and catalyst isospecificity.

Researchers have carried out experimental studies to elucidate the role of IDs in modulating the stereospecificity of catalytic systems, revealing two key principles [54,55,56]. First, IDs enhance stereoselectivity by transforming low-*isotactic* active centers into high-*isotactic* active centers, thereby increasing the fraction of high-*isotactic* active centers. Second, through steric interactions, IDs selectively occupy specific active centers, inhibiting their coordination with TiCl_4_ and effectively suppressing their catalytic activity in polymerization processes. This is the reason why the catalysts using 1,3-diether type IDs have higher stereoselectivity. Catalysts employing 1,3-diethers as IDs demonstrate remarkable stability upon exposure to AlEt_3_, thereby preserving their high stereoselectivity throughout the catalytic process [57,58]. In contrast, IDs containing carbonyl functionalities—particularly dialkyl phthalates—undergo partial leaching from the catalyst as a result of their chemical reactivity with the cocatalyst (AlEt_3_), which can compromise structural integrity and potentially reduce stereospecificity [38]. This leaching of IDs leads to a reduction in catalyst stereospecificity, as some isospecific active sites are converted into non-stereospecific ones. And the reason for the T_m_ depression is also obvious: the shortening of *isotactic* sequences in polymer chains, either due to more frequent steric errors or the steric error-induced chain termination to generate shorter polymer chains [56].

## 4. Conclusions

In this study, eight Z-N type catalysts for propylene polymerization were prepared via in situ reactions using distinct preparation methods. Systematic characterization and analysis were conducted on the crystal phase structure of the supports, as well as the morphology, composition, and particle size distribution of the catalysts. The catalytic performances of the eight catalysts (Cat-A-1, Cat-A-2, Cat-B-1, Cat-B-2, Cat-C-1, Cat-C-2, Cat-D-1, Cat-D-2) were evaluated through slurry polymerization. Further in-depth analyses of the resulting polypropylene samples were performed using ^13^C-NMR, DSC, and PP fractionation techniques to assess the stereoselectivity differences among the catalyst active sites. The key findings are summarized as follows:

(1) Catalyst Physicochemical Properties

The morphology, particle size, and crystal structure of Z-N type catalysts are determined primarily by the preparation method, not the ID. Specifically, the dissolution–precipitation method influences catalyst morphology and particle size more manifestly than the chemical conversion method. In contrast, the chemical composition of the catalyst is particularly its Ti content, which is largely governed by the ID’s chemical nature. Catalysts employing BMMF ID exhibit substantially lower Ti content than those using DNBP. XRD analysis confirmed that the ID significantly affects support formation. Compared to α-MgCl_2_, the catalyst support’s diffraction peaks showed distinct alterations: high-intensity peaks from the (003), (104), and (110) planes broadened, while low-intensity peaks corresponding to (103), (107), and (113) the vanished planes.

(2) Catalyst Activity and Stereoselectivity

Catalytic activity is strongly influenced by both the preparation method and the choice of ID, with a synergistic effect observed between these two factors. Under identical preparation conditions, catalysts based on BMMF ID exhibit substantially higher activity than their DNBP-based counterparts. The highest activity is achieved when BMMF ID is paired with the dissolution-precipitation method, attributable to two key mechanisms: (i) the chemical inertness of BMMF towards the co-catalyst during polymerization minimizes deactivation pathways, and (ii) this combination yields catalysts with the smallest particle size, thereby maximizing the exposure and accessibility of active Ti sites. In terms of stereoselectivity, BMMF-based catalysts also demonstrate superior performance compared to DNBP-based systems. This enhancement stems from both the inherent chemical stability of BMMF and the coordination of its ether oxygen atoms with Mg atoms adjacent to Ti active sites on the MgCl_2_ (110) surface. This coordination, combined with the steric hindrance of the BMMF substituents, reinforces spatial confinement around the Ti centers, resulting in improved control over chain propagation and consequently higher polypropylene stereoregularity.

(3) Polypropylene Properties

The M_w_, MWD, and T_m_ of PP are chiefly controlled by the type of ID used. When BMMF serves as the ID, the resulting PP exhibits a slightly lower molecular weight and a narrower MWD. Furthermore, PPs produced with BMMF ID-based catalysts show higher [mmmm] content, Tm, and increased C7-Ins fraction compared to PPs from DNBP ID-based catalysts.

This study was limited to laboratory-scale catalyst preparation; consequently, the applicability of the derived principles to kilogram-scale synthesis is considered for further expanding the current study to a practical technology-related development. Furthermore, given the inherent complexity of Z-N catalyst systems, future work should involve a multi-technique analytical approach, such as using STEM-EDS mapping and XPS depth profiling, to characterize the distribution of titanium on catalyst surfaces and within pores. Additionally, the number of effective active centers could be quantified via a quenching method. Further studies are currently underway.

## Figures and Tables

**Table 1:** Chemical composition and RSF of the pre-catalysts.

Catalysts	Support	Internal Donor	Ti% ^a^	Mg% ^b^	ID% ^c^	D_50_ μm	RSF
Cat-A-1	Mg(OEt)_2_ D_50_ = 25.61 μm	DNBP	2.76	18.67	15.4	25.80	0.882
Cat-A-2		BMMF	3.06	17.65	14.8	27.43	0.840
Cat-B-1	MgCl_2_·2.5EtOH D_50_ = 47.34 μm	DNBP	2.64	17.46	15.9	48.13	0.912
Cat-B-2		BMMF	3.13	16.88	14.2	49.26	0.929
Cat-C-1	δ-MgCl_2_ D_50_ = 210 μm	DNBP	2.25	19.12	16.2	25.55	0.827
Cat-C-2		BMMF	2.67	17.21	15.6	25.47	0.851
Cat-D-1	δ-MgCl_2_ D_50_ = 210 μm	DNBP	2.23	19.79	16.3	21.52	0.835
Cat-D-2		BMMF	2.90	17.78	15.3	20.68	0.896

^a^ The weight percentage of Ti in catalyst. ^b^ The weight percent of Mg in catalyst. ^c^ The weight percent of internal donor in catalyst.

**Table 2:** Influence of internal donor and preparation methods on pre-catalyst ^a^.

Catalysts	Internal Donor	CE ^b^ (g_pp_/g_cat_)	CA ^c^ (g_pp_/g_Ti_·h)	II ^d^ (%)	M_w_ × 10^5 e^ (g/mol)	*Ð* ^e^	T_m_ ^f^ (°C)	χ_c_ ^f^ (%)
Cat-A-1	DNBP	152	4903	97.54	2.86	7.68	161.0	46.2
Cat-B-1	DNBP	213	6310	98.06	2.55	7.49	161.8	47.7
Cat-C-1	DNBP	174	7733	98.05	2.53	6.94	163.3	47.0
Cat-D-1	DNBP	158	7085	97.09	2.63	6.83	160.2	46.3
Ref-1 ^h^	DNBP	32 × 10^3^	-	3.0	2.66	6.8	-	-
Cat-A-2	BMMF	167	5577	97.93	2.04	4.72	163.5	48.1
Cat-B-2	BMMF	259	6466	98.85	1.98	5.19	165.4	50.4
Cat-C-2	BMMF	239	8938	98.23	2.63	4.87	163.8	47.1
Cat-D-2	BMMF	269	9284	97.31	2.08	4.78	160.6	46.7
Cat-D-2 ^g^	BMMF	287	9896	98.12	2.33	4.98	161.5	46.5

^a^ Propylene polymerization condition: 15 mg precatalysts, T = 50 °C, t = 1 h, [Al]/[Ti] = 300 (mol/mol), c(TEA) = 2 mol/L, [Si]/[Ti] = 20 (mol/mol), c(CHMMS) = 0.05 mol/L. ^b^ Catalytic efficiency. ^c^ Catalytic activity. ^d^ Isotacticity index. ^e^ Weight-average molecular weight (M_w_) and molecular weight distribution (*Ð*). ^f^ Melting point (T_m_) and crystallinity (χ_c_). ^g^ T = 70 °C. ^h^ Ref-1 was prepared by dissolution-reprecipitation method.

**Table 3:** ^13^C-NMR Stereo-sequence distribution and MSL of polypropylene.

Polmer	mmmm	mmmr	rmmr	mmrr	rmrm	rrrr	mrrr	mrrm	MSL
Cat-A-1	88.46	3.52	1.67	1.39	0.81	0.81	0.71	1.16	20.5
Cat-A-2	90.23	2.13	1.02	2.07	0.83	0.63	0.53	1.05	28.9
Cat-B-1	89.29	2.85	1.38	1.89	0.69	0.88	0.52	0.97	23.9
Cat-B-2	93.05	2.36	0.49	1.32	0.65	0.58	0.32	0.61	41.3
Cat-C-1	92.27	1.73	1.11	1.59	0.60	0.63	0.46	0.73	31.3
Cat-C-2	92.89	1.65	1.54	0.71	0.66	0.58	0.37	0.76	35.2
Cat-D-1	87.85	3.92	1.86	1.69	0.93	0.86	0.83	0.49	18.6
Cat-D-2	89.35	2.41	1.87	2.33	0.69	0.64	0.48	1.02	23.5

## Data Availability

The original contributions presented in this study are included in the article. Further inquiries can be directed to the corresponding author.
